# Embedding and Chemical Reactivation of Green Fluorescent Protein in the Whole Mouse Brain for Optical Micro-Imaging

**DOI:** 10.3389/fnins.2017.00121

**Published:** 2017-03-14

**Authors:** Yadong Gang, Hongfu Zhou, Yao Jia, Ling Liu, Xiuli Liu, Gong Rao, Longhui Li, Xiaojun Wang, Xiaohua Lv, Hanqing Xiong, Zhongqin Yang, Qingming Luo, Hui Gong, Shaoqun Zeng

**Affiliations:** ^1^Britton Chance Center for Biomedical Photonics, Wuhan National Laboratory for Optoelectronics-Huazhong University of Science and TechnologyWuhan, China; ^2^Key Laboratory of Biomedical Photonics of Ministry of Education, Department of Biomedical Engineering, Huazhong University of Science and TechnologyWuhan, China

**Keywords:** embedding, chemical reactivation, fluorescent proteins, whole mouse brain, micro-imaging

## Abstract

Resin embedding has been widely applied to fixing biological tissues for sectioning and imaging, but has long been regarded as incompatible with green fluorescent protein (GFP) labeled sample because it reduces fluorescence. Recently, it has been reported that resin-embedded GFP-labeled brain tissue can be imaged with high resolution. In this protocol, we describe an optimized protocol for resin embedding and chemical reactivation of fluorescent protein labeled mouse brain, we have used mice as experiment model, but the protocol should be applied to other species. This method involves whole brain embedding and chemical reactivation of the fluorescent signal in resin-embedded tissue. The whole brain embedding process takes a total of 7 days. The duration of chemical reactivation is ~2 min for penetrating 4 μm below the surface in the resin-embedded brain. This protocol provides an efficient way to prepare fluorescent protein labeled sample for high-resolution optical imaging. This kind of sample was demonstrated to be imaged by various optical micro-imaging methods. Fine structures labeled with GFP across a whole brain can be detected.

## Introduction

Resin embedding has been widely applied in light and electron microscopy analysis of biological samples (Newman et al., 1949; Mikula et al., 2012; Mikula and Denk, 2015). This technique hardens tissue to facilitate ultra-thin sectioning. Combined with optical imaging techniques, we can acquire optical images of tissue and then reconstruct the three-dimensional morphology of neurons in a whole brain at a sub-micron axial resolution (Li et al., 2010; Quan et al., 2016). Aequorea victoria green fluorescent protein (GFP) can be used to label specific proteins and subcellular compartments *in vivo*, which facilitates the study of many aspects of neuroscience (Tsien, 1998; Feng et al., 2000; Oh et al., 2014). Unfortunately, the procedure for dehydration and resin embedding weakens the fluorescent signals of GFP (Becker et al., 2012; Ragan et al., 2012), making the signals difficult to detect.

In recent years, many improvements have been made in the procedure for biological tissue embedding, such as cryofixation, dehydration, and embedding at low temperatures (Jorgensen and McGuffee, 1987; Luby-Phelps et al., 2003; Micheva and Smith, 2007; Nixon et al., 2009; Watanabe et al., 2011; Peddie et al., 2014). Until now, many methods were only used in cell or small tissue block studies, and it is not certain whether GFP fluorescence is well-preserved in the tissue using these methods. Other methods preserve the fluorescent signal to some extent by adjusting the pH value of the resin (Watanabe et al., 2011; Yang et al., 2013), but this approach greatly affects the resin polymerization and reduces the quality of cutting. Although the methods mentioned above allow for the detection of GFP signals, because fluorescence quenching still exists, researchers do not believe that it is feasible for GFP to be applied in resin embedding technology (Ragan et al., 2012). Therefore, it is still a challenge to improve the brightness of the fluorescent protein in a large volume of resin-embedded tissue.

**Figure 1:**
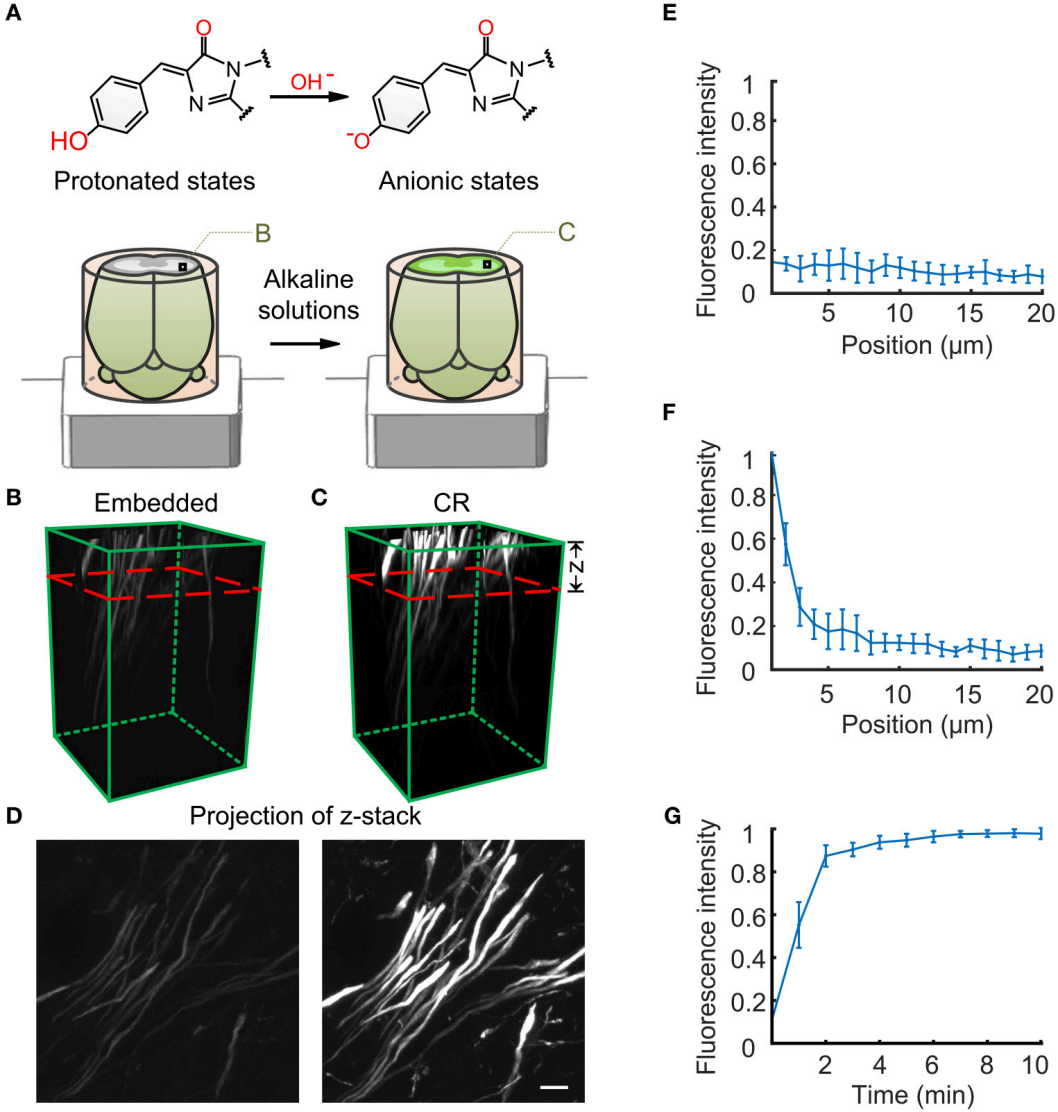
**The principle reaction of chemical reactivation for fluorescence imaging. (A)** Diagram of the course of GFP and chromophore changes in brain tissue during resin embedding and chemical reactivation. **(B)** The fluorescent state of nerve fiber tracts in the striatum region of resin-embedded thy1-GFPM mouse brain (left). **(C)** The fluorescent state reactivated by alkaline buffer solutions. **(C)** The red dashed line indicates the activation depth of alkaline buffer solutions. The z axis represents the alkaline buffer penetration depth. **(D)** Projection of the same z-stack from the imaging surface to the red dashed line in **B** and **C**. **(E)** Original fluorescence intensity distributions for the resin-embedded brain as a function of imaging depth. **(F)** Fluorescence intensity distributions after alkaline buffer activation for 2 min. **(G)** Fluorescence intensity distributions 1 μm below the surface of the resin-embedded brain over time. **(E–G)** The fluorescence intensity values are given as the mean ± SD (*n* = 6). Scale bars: **(B,C)**, 20 × 20 × 50 μm^3^; **(D)**, 2 μm.

To solve these problems, our lab systematically studied the mechanism of GFP molecule quenching in acrylic resin (Xiong et al., 2014). We found that GFP molecules are transformed into a non-fluorescent state because of the protonation of the GFP chromophore during resin embedding rather than by direct denaturation in acrylic resin. Under an alkaline environment, the protonated chromophore of GFP can be transformed into an anionic state, which results in the recovery of GFP fluorescence molecules in resin-embedded tissue because the GFP recovers its fluorescent state through the permeation of alkaline solutions (over a pH range of 9–12) (Figure 1). According to this mechanism, we propose a principle of chemical reactivation (CR) that can greatly enhance the fluorescence intensity of GFP and YFP in resin-embedded biological tissue.

**Figure 2:**
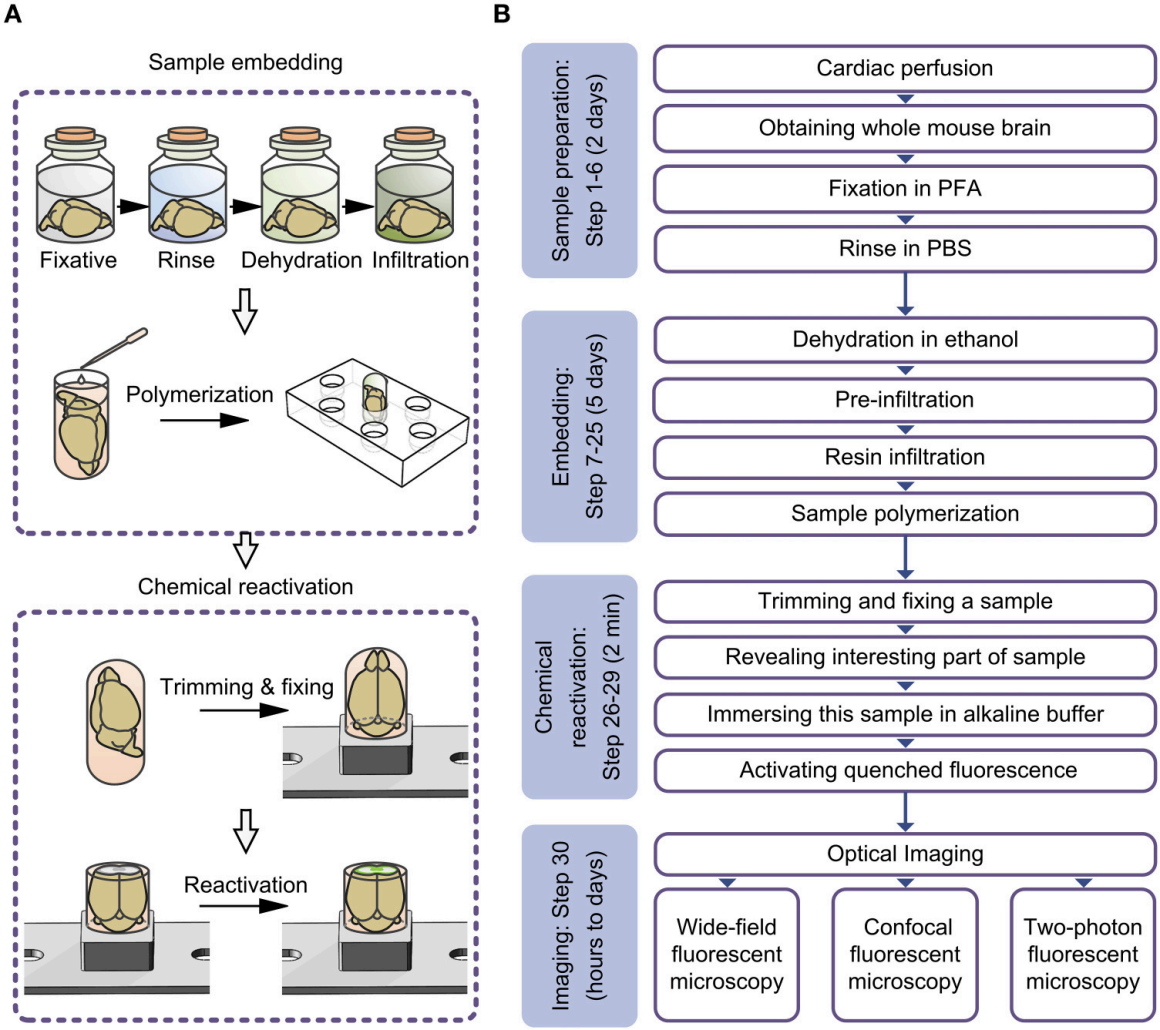
**Schematic diagram for the entire experimental procedure. (A)** Schematic diagram illustrating the procedure for embedding whole mouse brains and chemically reactivating the quenched fluorescence. **(B)** Flowchart for the all steps of the protocol.

The whole brain sample preparations involve multiple steps and demands stringent specification. Improper operation of any step may cause a decrease of sample quality or even failure. Sample preparation with stably quality and high efficiency requires a detailed and standard protocol to guide experimental operation. In this study, we provide a detailed protocol for resin embedding of a whole brain and the CR of fluorescent protein. This protocol includes two parts: the complete procedure for whole brain embedding and chemical reactivation of the fluorescent signal in resin-embedded tissue (Figure 2). This protocol can be employed to prepare biological tissue for good ultrathin section cutting, and to observe the fine structures of neural cells under a light microscope. This approach was used in our previous publication (Xiong et al., 2014; Gong et al., 2016), and in this article, we provide an optimized version of the procedure. Compared with our previous version, this procedure prevents the fluorescence reduction caused by treatment with complete dehydration and largely preserves the neural details labeled by GFP/YFP. This procedure is now a standard protocol in our lab.

### Applications and advantages of the method

In CR, the fluorescence signal of biological tissue can be greatly enhanced, even for ultrathin (70–200 nm) section from an HM20-embedded tissue. Therefore, the resin-embedding method is compatible with GFP and its variants fluorescence labeling techniques. This protocol can be applied for resin-embedding of biological tissue and well preserves the fluorescence signal and structure of the biological tissue. This protocol is compatible with many fluorescence imaging techniques, such as wide-field epifluorescence, confocal, and two-photon microscopy. The fine structures of neurons, such as the filopodium and axon terminal boutons, can be clearly observed in the resin-embedded brain through light microscopy. Combined with imaging and slicing, this protocol can be used to acquire full three-dimensional morphological structures of specifically labeled cell in a large volume of biological tissue. The resin-embedded sample can be preserved long-term, and its fluorescent signal can be maintained under −20°C and dark conditions for more than 2 year. This protocol can be applied to resin-embedded samples labeled by GFP, YFP, and GFP mutant strains. However, chemical activation has no obvious effect on embedded samples labeled with red fluorescent protein (mCherry and DsRed, etc.) or fluorescent dye (Alexa 647 and CY5, etc.) because they are not sensitive to protonation.

## Experimental design

### Sample selection

To ensure a better imaging result, the brain samples should be well-labeled either through a transgenic technique or virus labeling. If the fluorescent signal of the sample is too weak, it will reduce the imaging quality. before processing, the sample should be examined with a fluorescence microscope by a quick glimpse.

### Fixation

For histology, the purpose of fixation is to stabilize the *in vivo* tissue structure and protect it from the deleterious effects of dehydration, infiltration and embedding (Oliver and Jamur, 2010). Aldehyde fixatives can preserve the morphology and structure of cells (Schnell et al., 2012; Wu et al., 2012). In the screening experiments, we found that 2.5% glutaraldehyde enhanced the autofluorescence compared to 4% paraformaldehyde (PFA) in tissue. The autofluorescence can reduce the contrast of the images because the autofluorescence emission spectra overlap with GFP signals in brain tissue. Meanwhile, Add sucrose in PFA solution will help to maintain cell shape (Kusser and Randall, 2003). The possible reason is that the fixation process may result in partial water loss of tissue, which induce uneven shrinkage and deformation of the cell. Therefore, from our previous experience, we added 2.5% sucrose in 4% PFA solution to fix the brain tissue.

Fixation can be divided into cardiac perfusion fixation (step 3) and post-fixation (step 5). After perfusion fixation, the brain has certain hardness and can be more easily peeled away. Post-fixation can further increase the hardness of the brain, reduce cell autolysis and maintain cell structure (Paavilainen et al., 2010). In this process, the time and temperature will affect the fluorescence signal of the brain tissue. If the fixation time is too short, the brain will have serious deformations during the latter process of dehydration and resin embedding. In contrast, if the fixation time is too long, the fluorescence signals will be quenched and the autofluorescence in the tissue will be enhanced. A suitable fixation time for the whole mouse brain is 24 h at 4°C in the dark.

### Rinsing

Residual fixative can be removed by rinsing with phosphate-buffered saline. Therefore, complete rinsing can reduce the autofluorescence caused by PFA of a resin-embedded brain. Rinsing can enhance the contrast of the images, especially when detecting weak fluorescent labeling signals during imaging.

### Dehydration

**Figure 3:**
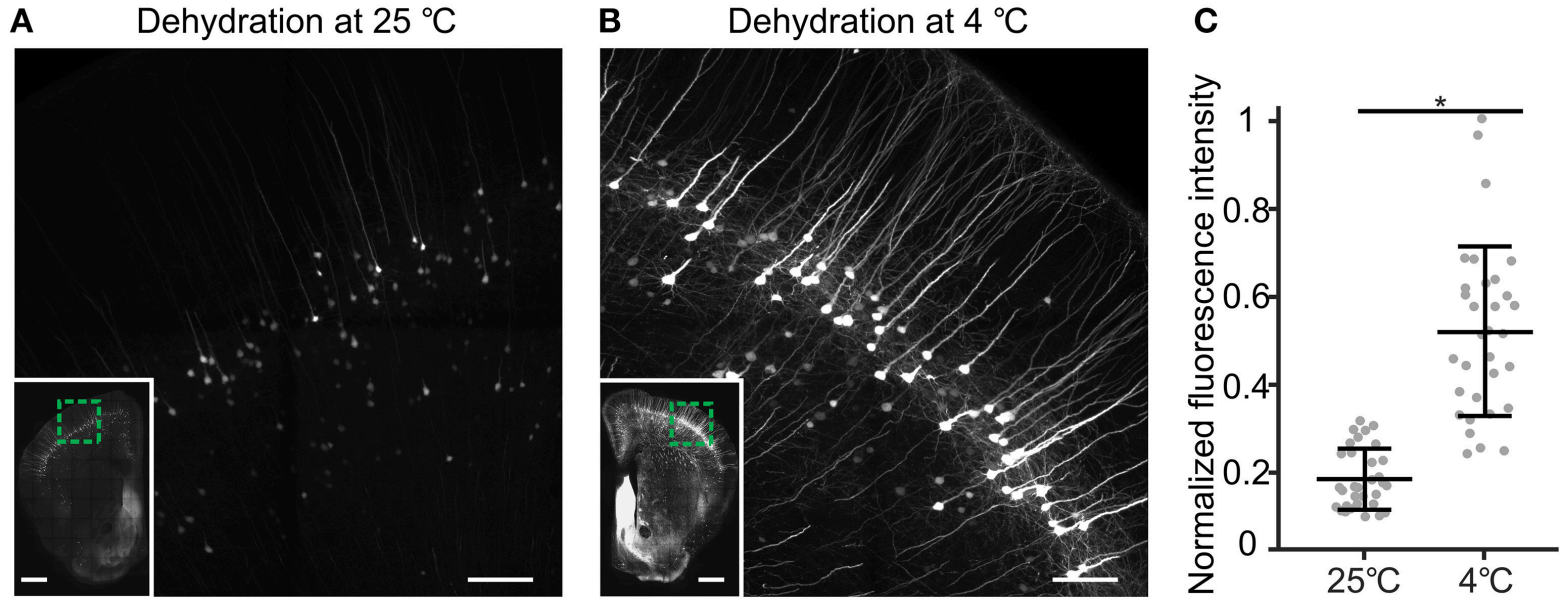
**The influence of dehydrating temperature on CR-induced fluorescence recovery. (A)** thy1-YFP H mouse brain was divided into the left hemisphere and the right hemisphere. The left and right hemispheres were dehydrated at 25 and 4°C, respectively, and then embedded in resin. After embedding, the same coronal plane area was revealed and immersed in 0.05 M Na_2_CO_3_ solution for 1 h. **(A)** Fluorescence brightness of the left hemisphere. **(B)** Fluorescence brightness of the right hemisphere. **(A,B)** Two regions of a single brain. The inset images in **(A,B)** show the same coronal plane area, and the green box indicates **(A,B). (C)** The normalized fluorescence intensities were comparable for the samples dehydrated at 25 and 4°C (mean ± SD; ^*^p < 0.01 in two-tailed *T*-test; *n* = 32 each). Scale bars: **(A,B)**, 100 μm; **(A,B)**, green box, 0.5 mm.

Generally speaking, resin cannot directly penetrate biological tissue because biological tissue contains a large quantity of water, and resin is not soluble with water at any proportion (Newman and Hobot, 2012). To force the resin to infiltrate the tissue, we required organic solvents that are miscible with resin and water to remove water from the tissue. Through tissue dehydration, water is removed from the tissue by adding increasing concentrations of organic solvent. Then, the resin can evenly infiltrate the internal tissue (Echlin, 2009). Acetone and ethanol are typical dehydrating agents (McDonald, 2014). In our process, acetone dehydration was faster than ethanol dehydration, and there was no difference between the solutions in fluorescence preservation. However, because oxygen and acetone can hinder acrylic resin polymerization (Suvarna et al., 2013), acetone results in incomplete or a lack of polymerization in acrylic resin (Watanabe and Jorgensen, 2012). Thus, we chose ethanol as the gradient dehydrating agent for whole mouse brains. Additionally, the temperature at which dehydration is performed is very important for fluorescence preservation. Through testing, we found that fluorescence could be preserved well in ethanol at 4°C. However, the fluorescence was irreversibly quenched by ethanol at room temperature (25°C; Figure 3). At a temperature of 4°C, dehydration times were set depending on the size of the sample. For example, dehydration of a whole mouse brain requires 12 h, while dehydration of half a brain requires only 6 h.

### Selection of resin types

For embedding fluorescence-labeled tissue, many characteristics of embedding agents should be considered, including autofluorescence, viscosity, hydrophilic-hydrophobic properties, polymerization type, mechanical properties, and other factors. Epoxy resin has a good cutting performance, whereas it is difficult for epoxy to penetrate tissue because of its high viscosity. Acrylic resin has a low viscosity, can easily penetrate tissue and has low autofluorescence, which can be optimal for optical imaging. Furthermore, acrylic resin is also superior to epoxy resin for fluorescence preservation.

Acrylic resin materials were divided either into hydrophilic and hydrophobic groups according to their hydroxyl groups or into cross-linking and non-cross-linking groups according to their double functional groups. Hydrophilic resin, such as Lowicryl K4M, GMA, or LR White resin, can tolerate a small amount of water in polymerization, at a mass ratio of ~10–12% (Newman and Hobot, 2012). For polymerization of hydrophobic resins, such as MMA, Lowicryl HM20, and Lowicryl HM23, the tissue must be completely dehydrated (Newman and Hobot, 1999); otherwise, it is very difficult for the resin to evenly penetrate tissue. Cross-linking resins, such as Lowicryl K4M, LR White, and Lowicryl HM20, have very good cutting performance and can produce nanoscale sections of tissue. In the sectioning process, the slice is not curly and is easier to collect than those obtained with non-cross-linking resin. In contrast, the cutting performance of non-cross-linking resins, such as GMA and MMA, is poor. In addition, when cutting ultrathin sections, the tissue can easily break loose from the surrounding resin.

Lowicryl HM20 resin, with its low viscosity, can quickly complete penetration into a large volume of biological tissue. At the same time, its low autofluorescence and its ability to preserve ultrastructure will ensure imaging quality (Nanguneri et al., 2012). Taking all of these advantages into account, we chose HM20 resin as the embedding medium for our protocol.

### Resin permeability and polymerization

The infiltration time for a sample depends on the sample size, resin viscosity, and temperature. At 4°C, the rate of resin infiltration is greater compared to temperatures below zero. Through testing, we found that the complete infiltration time for a mouse brain is ~3 days.

Polymerization methods for acrylic resin include heat polymerization, catalytic polymerization, and UV polymerization. Because it is difficult for UV light to penetrate thick tissue, UV polymerization is typically used when embedding small volumes of tissue (Newman and Hobot, 1999). Catalytic polymerization is very easily inhibited by oxygen and often causes incomplete polymerization of the resin, and in our study, the results were not consistent. Therefore, catalytic polymerization and UV polymerization were not suitable for embedding tissue at a large volume (Schwarz and Humbel, 2007). Heat polymerization at 50°C to 60°C produced reproducible results when benzoyl peroxide (BPO) was used as an initiator. However, we found that the fluorescence was quenched substantially at temperatures above 55°C. If the polymerization were performed in a vacuum environment, the sample could be completely polymerized at 50°C.

### Chemical reactivation

Based on the previous test results, we found that CR could be applied to many types of acrylic resins, such as LR white, GMA, Technovit 9100, and Lowicryl HM20. For hydrophilic embedding media (LR White and GMA), 2 min of alkaline liquor penetration can enhance fluorescence in a 9-μm-thick layer, but the penetrated layer was only 4 μm thick when using a hydrophobic medium (Technovit 9100 and Lowicryl HM20). In theory, basic chemical molecules or ion groups which hydrolyze hydroxyl ions can produce a chemical activation effect for resin-embedded tissue labeled by GFP, YFP, or other GFP mutants. The basic chemical molecule or ion group will hydrolyze hydroxyl ions, which then activate the fluorescence of GFP. Typical basic solutions have pH values in the range of 9–12, such as sodium hydroxide solution, ammonia solution, and ethylene diamine solution (Xiong et al., 2014). We used 0.05 M Na_2_CO_3_ as an alkaline buffer solution because it is less abrasive, non-toxic and non-irritating to the human body.

## Materials

### Tissue

Thy-1 EGFP M line (JAX stock number 007788) and Thy-1 YFP H line (JAX stock number 003782) transgenic mice, separately expressing GFP and YFP tag in the whole brain were used in this protocol. All of the mouse procedures were approved by the Institutional Animal Ethics Committee of the Huazhong University of Science and Technology.

### Fixative and perfusion reagents

- Chloral hydrate.
- Phosphate buffered saline (PBS) powder, pH 7.4 (Sigma-Aldrich, cat. no. P3813-10PAK).
- PFA (Sigma-Aldrich, cat. no. P 6148-1KG).

### Dehydration reagents

- Ethyl alcohol (absolute, 200 proof).

### Pre-infiltration reagent

- Ethyl alcohol (absolute, 200 proof).
- Lowicryl HM20 Resin Kits (Electron Microscopy Sciences, cat. no. 14340).

### Infiltration reagent

- Lowicryl HM20 Resin Kits (Electron Microscopy Sciences, cat. no. 14340).

### Polymerization reagents

- Lowicryl HM20 kit (Electron Microscopy Sciences, cat. no. 14340).
- Dibenzoyl peroxide (98%, J&KChemical, cat. no. 934368).

**ΔCRITICAL STEP:** Note that we did not use the Lowicryl HM20 kit initiator-C in this protocol because it is used for UV photo-initiated polymerization. Instead of initiator-C, we use BPO initiate resin polymerization.

### Chemical reactivation reagents

- Sodium carbonate (anhydrous, Sigma-Aldrich, cat. no. 222321-500G).

### Equipment

**Figure 4:**
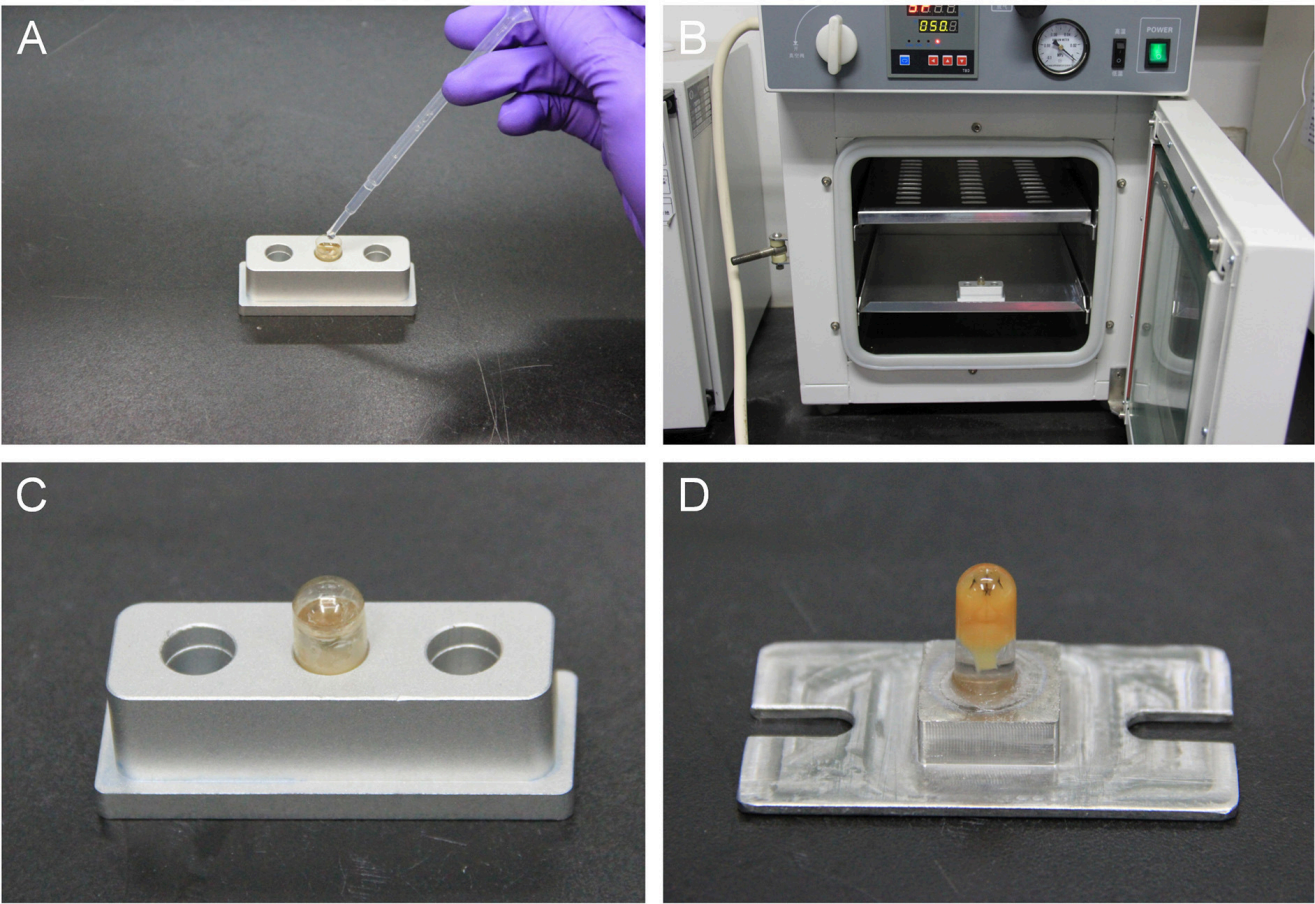
**The steps for polymerization. (A)** Place the gelatin capsule in the hole of an aluminum base used for heat conduction when polymerizing samples. Then, transfer the brain into the gelatin capsule and immediately cover the lid of the gelatin capsule. **(B)** Place the sample in the oven. **(C)** At the end of polymerization, remove the sample from the oven. **(D)** Glue the sample onto a base plate.

- pH meter.
- Buchner funnels (200–300 mesh).
- Thermostatic water bath.
- Vibration microtome (Leica, VT1000 S).
- 000# gelatin capsule (Electron Microscopy Sciences, cat. no. 130-12).
- Aluminum capsule base (Figure 4A).
- Imaging software (http://rsbweb.nih.gov/ij/).

### Microscopes and imaging system

- Confocal microscope (Zeiss, LSM710; LSM780; Nikon A1).
- Wide-field fluorescence microscopy (Nikon, Ni-E).
- Two-photon fluorescence micro-optical sectioning tomography (2 P-fMOST) system.

## Reagent setup

### Fixative and perfusion

#### Phosphate buffer (0.01 M)

Dissolve a pouch of 0.01 M PBS powder in 1 l of distilled water and adjust the pH to 7.4. Store this solution at 4°C for up to 1–2 months.

#### Chloral hydrate (5%, wt/vol)

Dissolve 5 g chloral hydrate in 100 ml 0.9% normal saline (NS) solution with sufficient mixing. Store this solution at 4°C in the dark for up to 6 months. **CRITICAL:** Stirring to dissolve chloral hydrate at room temperature without heating is necessary to prevent the chloral hydrate from being decomposed.

#### PFA (4%, wt/vol)

Add 40 g PFA and 25 g sucrose to 1 l 0.01 M PBS. Heat and stir the mixture in a 60°C water bath. The PFA powder can be dissolved in ~1 h. Then, cool the solution to room temperature and remove the residual with a Buchner funnel. Label the tube clearly. CRITICAL: After cooling, the PFA powder might not be dissolved completely, the residual in PFA solution should be removed by Buchner funnel. **! CAUTION:** PFA is a strong irritant and a toxic reagent, and it is harmful to human health. Avoid inhalation or exposing eyes or skin directly to PFA. Wear gloves, a mask and goggles to handle PFA in a fume hood.

### Dehydration

#### Absolute ethyl alcohol

Transfer 50 ml of the stock chemical into a centrifuge tube (50 ml). Screw the lid on tightly and keep the tube sealed. Label the tube clearly.

#### Graded ethanol solutions

Prepare 50, 75, and 95% (vol/vol) graded ethanol solutions in distilled water. For example, for 50% ethanol solutions, add 1 volume absolute ethyl alcohol to 1 volume distilled water in a centrifuge tube. Screw the lid on tightly and shake the container for a few minutes. Prepare other graded ethanol solutions and label the tubes clearly.

### Pre-infiltration

#### Lowicryl HM20 kits

Lowicryl HM20 kits include three components: monomer mixtures-E, cross-linker-D, and a photo-initiator-C. The photo-initiator was not used to initiate polymerization in this protocol. The monomer mixture-E and cross-linker-D consisted of monomethyl ether hydroquinone (MEHQ), which is used as a polymerization inhibitor. This inhibitor can quench GFP and GFP-variant fluorescence, inhibit initiator decomposition generating free radicals, and affect the quality of resin polymerization. **CRITICAL:** The filtered Lowicryl HM20 kit should be stored at 4°C in the dark.

### Filtered resin monomer mixtures and cross-linker

Place a small amount of cotton wool into a chromatography column outlet. The chromatography column should be filled with 3/4 aluminum oxide powder. Then, fix the column on a retort stand and add the resin monomer mixtures. Seal the plug and let nitrogen pass through the chromatography column slowly to destabilize the monomer mixtures. A column filling with Al_2_O_3_ can destabilize 500 g of monomer mixtures. Place the destabilized monomer mixture in a sealable brown glass bottle and store the mixture at 4°C. Filter and store the cross-linker according to the following steps. Label the tube clearly. **! CAUTION:** Lowicryl HM20 kits contain irritants and toxic components and can cause allergies. Avoid inhalation or direct exposure to skin or eyes. Wear nitrile gloves, a mask and goggles to handle all components of the Lowicryl HM20 resin in a fume hood.

### Resin working solution

Weigh 0.2 g of BPO, 33 g of monomer mixture and 7 g of cross-linker and place in 60 ml sealable brown glass bottles. Add a magnetic stir bar and gently stir for 10 min or by bubbling dry nitrogen into a resin solution until the BPO completely dissolves. Screw the lid on tightly and keep the bottle sealed. Label the bottle clearly and store it at 4°C. **CRITICAL:** BPO particles can slowly decompose at room temperature. The bottle should be sealed and stored at −8°C. When preparing working solutions, BPO should be added prior to the monomer mixture and cross-linker to prevent BPO particles from sticking to the wall of the bottle. The stirring time should not be long to prevent excess oxygen from incorporating into the resin. Seal the bottle and prevent exposure to aqueous vapor when stored at 4°C. Oxygen and aqueous vapor will affect resin polymerization. **! CAUTION:** BPO is an irritant, is toxic, and can cause allergies. Avoid inhalation or direct exposure to skin or eyes. Wear nitrile gloves, a mask and goggles when handling BPO and all components of the Lowicryl HM20 resin in a fume hood.

### Pre-infiltration solutions

Prepare 1:1 and 2:1 (vol/vol) resin pre-infiltration solutions in absolute ethanol. For example, for a 1:1 resin pre-infiltration solution, add 1 volume resin working solution to 1 volume absolute ethanol in a sealable brown glass bottle. Screw the lid on tightly and shake it gently. Prepare the 2:1 resin pre-infiltration solution according to the following steps. Label the bottle clearly and store it at 4°C. **CRITICAL:** This solution cannot be used repeatedly, and avoid long-term storage. **! CAUTION:** Wear nitrile gloves, a mask, and goggles to handle all components of Lowicryl HM20 resin in a fume hood.

### Infiltration solution

Transfer 10 ml of resin working solution into 20 ml sealable brown glass bottles. Screw the lid on tightly and keep the bottle sealed. Label the bottle clearly and store it at 4°C. **! CAUTION:** Wear nitrile gloves, a mask, and goggles to handle all components of Lowicryl HM20 resin in a fume hood.

### Chemical reactivation solution

Prepare 1 l of 0.05 M Na_2_CO_3_ buffer. Weigh 5.3 g of anhydrous sodium carbonate and add to 1 L of distilled water; stir the solution well to ensure that the sodium carbonate powder completely dissolves.

## Procedure

### Tissue preparation • timing 1 h

1. Preheat 0.01 M PBS (pH 7.4) to 37°C in a water bath.
2. Anesthetize the animal using chloral hydrate (0.5%, wt/vol) in an intraperitoneal injection at 0.1 ml/g body weight.
3. Perform cardiac perfusion using the preheated 0.01 M PBS for 10 min at a perfusion rate of 9 ml/min. When the liver is bleached, exchange the PBS for PFA (4%, wt/vol) for 15 min at a perfusion rate of 6 ml/min.

**ΔCRITICAL STEP:** For mice younger than 6 weeks, adjust the PFA perfusion rate to 4 ml/min because a higher PFA perfusion rate can result in tissue distortion.

## ? Troubleshooting

4. Carefully remove the whole brain from the skull.

**ΔCRITICAL STEP:** Take care not to injure the pallium, and keep the brain and spinal cord integrated. Do not allow the surface of the brain to become dry.

**! CAUTION:** Perform all steps with PFA in a fume hood and wear nitrile gloves, a mask, and goggles.

## ? Troubleshooting

### Fixation • timing 1 day

5. Immediately immerse the brain samples in PFA (4%, wt/vol) for 24 h, and store them at 4°C, away from light.

**ΔCRITICAL STEP:** Avoid over-fixation of the brain samples. The fixation time should not be more than 24 h; otherwise, fixation can enhance the tissue autofluorescence.

**! CAUTION:** waste PFA solution must be collected in appropriate waste containers.

## ? Troubleshooting

### Rinsing • timing 1 day

6. After fixation, exchange three washes of precooled 0.01 M PBS (pH 7.4) for PFA solution (4%, wt/vol) in a 4°C refrigerator with 50 ml for each group; allow 6 h for the first wash, 6 h for the second wash, and 12 h for the third wash.

## ? Troubleshooting

### Dehydration • timing 1 day

7. Place the brain in a centrifuge tube (50 ml), and add 50% ethanol solution using a Pasteur pipette three times, incubating the samples for 1 h each time; close the lid of the tube immediately.
8. Cover the centrifuge tube with aluminum foil to keep the samples in the dark, and place the tube in a 4°C refrigerator.
9. Replace 50% ethanol with 75, 95%, and absolute ethanol solution, respectively. Dehydrate the samples with each solution for 2 h.
10. Change the absolute ethanol solution three times, and incubate the samples for 2 h each time.

**ΔCRITICAL STEP:** Keep the centrifuge tubes sealed to prevent aqueous vapor from entering. Store all dehydration solutions at 4°C in the dark.

## ? Troubleshooting

### Pre-infiltration • timing 4 h

11. Transfer the brain from an absolute ethanol solution to a 10 ml brown glass bottle, and immediately add 50% pre-infiltration solution that was prepared in advance (50% ethanol/50% resin working solution); then, close the lid of the glass bottle.

**! CAUTION:** Handle all pre-infiltration steps in a fume hood and wear nitrile gloves, a mask, and goggles.

12. Store the sample in a 4°C refrigerator for 2 h.

**ΔCRITICAL STEP:** The samples should be stored at 4°C in the dark after each step.

13. Discard the 50% pre-infiltration solution in appropriate waste containers, and add the 75% pre-infiltration solution (25% ethanol/75% resin working solution) into glass bottle, close the lid of the glass bottle.
14. Repeat step 8 to complete the procedure for pre-infiltration.

**! CAUTION:** waste pre-infiltration solution must be collected in appropriate waste containers.

### Infiltration • timing 3 days

15. Switch out the pre-infiltration solution with the resin infiltration solution in a 10 ml brown glass bottle, and close the lid of the glass bottle tightly. Seal the lid with poly(tetrafluoroethylene) tape (PTFE tape).

**! CAUTION:** Handle all infiltration steps in a fume hood and wear nitrile gloves, a mask, and goggles.

## ? Troubleshooting

16. Store the samples in a 4°C refrigerator for 2 h.

**ΔCRITICAL STEP:** All infiltration steps should be conducted at 4°C in the dark.

17. Replace the original solution with 10 ml of fresh infiltration solution, and close the lid of the glass bottle tightly. Seal the lid with PTFE tape.
18. Store the sample in a 4°C refrigerator for 24 h.
19. On the next day, repeat steps 17–18.
20. On the third day, repeat steps 17–18. Complete the procedure for resin infiltration.

**ΔCRITICAL STEP:** To ensure optimal polymerization, the infiltration solution cannot be reused. All solutions for infiltration should be kept at 4°C in the dark. **! CAUTION:** Waste infiltration solution must be collected in appropriate waste containers.

## ? Troubleshooting

### Polymerization • timing 12 h

21. After the infiltration process, take the brain sample from the 4°C refrigerator and warm to room temperature; then, wipe any water drops from the bottle wall.

**! CAUTION:** Handle all polymerization steps in a fume hood and wear nitrile gloves, a mask, and goggles.

22. Open a 000# gelatin capsule, and place it on an aluminum base (Figure 4A). Then, transfer the brain into the gelatin capsule using ophthalmic forceps.

**ΔCRITICAL STEP:** Use Aluminum base can prevent an uncontrolled rise in polymerization temperature, and stabilize polymerization rate.

## ? Troubleshooting

23. Fill the gelatin capsule with infiltration solution using a Pasteur pipette (Figure 4A). Close the capsule immediately.

**ΔCRITICAL STEP:** Old infiltration solution applied to the brain sample is not used for polymerization. You should employ new infiltration solution to polymerize the brain sample.

24. Place the sample in a sealed drying vacuum oven (Figure 4B). Maintain the temperature at 50°C for 12 h.

## ? Troubleshooting

25. Remove the resin block from the drying vacuum oven (Figure 4C) and carefully strip away the capsule.

### Trimming and fixing • timing 20 Min

26. Trim the block using a filer and adhere it to a sample base plate, ensuring that the brain is in the center (Figure 4D).

**! CAUTION:** When trimming the resin block, the trimming will produce resin chippings that can result in allergies. Avoid inhalation or direct contact with skin and eyes. Wear nitrile gloves, a mask and goggles during this procedure.

27. Place the sample on a miller and expose the part of the brain being examined.

### Chemical reactivation • timing variable (typically 2 Min)

The Depth of chemically reactivated layer can vary depending on alkaline liquor penetration time. Two minutes of penetration can fully activate 1 μm below the surface of the resin-embedded brain and produce an enhanced fluorescence layer ~4 μm thick (Figures 1E–G).

28. Add 0.05 M Na_2_CO_3_ buffer (pH = 11.4) to a sink and immerse the sample in it.

## ? Troubleshooting

29. Two min of 0.05 M Na_2_CO_3_ buffer immersion enhances the fluorescence layer for a sample surface that is ~4 μm thick. One min of immersion can result in a ~90% fluorescence recovery for 1 μm below the surface of the sample. With prolonged immersion time, the thickness of the fluorescence layer is continuously increased.

### Imaging • timing ~30 Min

30. After the fluorescence of the sample surface is activated by 0.05 M Na_2_CO_3_ buffer, use confocal, two-photon or a wide-field microscopy to examine the sample.

## ? Troubleshooting

Troubleshooting advice can be found in Table 1.

**Table 1:** **Troubleshooting table**.

**Step**	**Problem**	**Possible reason**	**Solution**
3	Brain with strong spontaneous fluorescence	Too much blood residue in the brain	Prolong the time of PBS perfusion
	A cavity in the ventricle of a resin-embedded brain	Bubble entered the ventricle during PBS/PFA perfusion	Check and drain the bubble in a conduit
	Morphological changes in neurons in the brain tissue	Mouse is too young or perfusion rate is too high	Use an adult mouse or reduce the rate of perfusion
4	Weak or no fluorescence signal	Expression of fluorescent signal is too weak	Check whether the signal can be detected by confocal microscopy or change to another method of fluorescence labeling
5	Morphological changes in neurons in the brain tissue	PFA post-fixation time is too short	Prolong the post-fixation time to 24 h
6	Brain with strong spontaneous fluorescence	PFA post-fixation time is too long	Shorten post-fixation time to 24 h
		PBS rinsing time is too short	Prolong the rinsing time
7–10	Weak or no fluorescence signal	Dehydration temperature is too high	Dehydrate the sample at 4°C
	Morphological changes in neurons in the brain tissue	Dehydration occurs too rapidly	Dehydrate in an ethanol gradient
10	A cavity in the center of a resin-embedded sample	Incomplete dehydration	Use unopened ethanol and prepare a new graded series of ethanol solutions
15	Bubbles appear in the resin block	Use of too much initiator	Accurately weigh the initiator
	The sample was not fully polymerized	Initiator failed	Use a new initiator
		Oxygen mixed into the resin	Perform the polymerization in vacuum
20	A cavity in the center of a resin-embedded sample	Infiltration time is <3 days	Prolong infiltration time to 3 days
22	The sample was not fully polymerized	Breakage of gelatin capsule	Check the gelatin capsule before polymerization of the sample
24	Bubbles appear in resin block	Initial polymerization temperature is too high	Set initial polymerization temperature to 50°C
		The temperature of the oven is not stable or the oven is not under vacuum	Check the temperature and vacuum state of the oven
	Morphological changes in neurons in the brain tissue	Rate of polymerization is too high	Initiate resin polymerization at 50°C
28	Weak or no fluorescence signal	CR solution is invalid or the pH is below 9	Measure and adjust the pH of the alkaline solution to be above 9

### • Timing

Steps 1–4, tissue preparation: 1 h

Steps 5, fixation: 1 d

Steps 6, rinse: 1 d

Steps 7–10, dehydration: 1 d

Steps 11–14, pre-infiltration: 4 h

Steps 15–20, infiltration: 3 d

Steps 21–25, polymerization: 12 h

Steps 26–27, trim and fix: 20 min

Steps 28–29, chemical reactivation: ~2 min

Steps 30, imaging: ~30 min

## Anticipated results

The quality and fluorescent signals of resin-embedded samples can be examined when the resin has polymerized. After the resin polymerization, there should be no air bubbles in the sample. If the rate of resin polymerization is too high, bubbles will form in the resin (see Supplementary Figures 1A,B), and the samples may become brittle. Rapid polymerization causes poor cutting performance. When a cavity appears in the center of a sample, it indicates that the brain was not completely dehydrated or infiltrated (see Supplementary Figures 1C,D). However, if the cavity is only located in a ventricle, it means that air has perfused into a ventricle during the procedure of cardiac perfusion because air will hinder the infiltration of resin into the ventricle (Supplementary Figure 2).

The dehydration temperature is critical for fluorescence preservation, and dehydration at room temperature can cause irreversible fluorescence quenching. Through testing, we found that brains can be rapidly dehydrated at 4°C with good fluorescence preservation (Figure 4). Whole-brain embedding involves the processes of fixation, dehydration, and embedding. Problems with any of the steps discussed above can lead to a morphological distortion of the neurons. If all procedures are strictly followed, one possible reason for problems is that the mouse was too young (<4 weeks) or the PFA perfusion rate was too high. For immature mice, the PFA perfusion rate should be adjusted to 4 ml/min.

**Figure 5:**
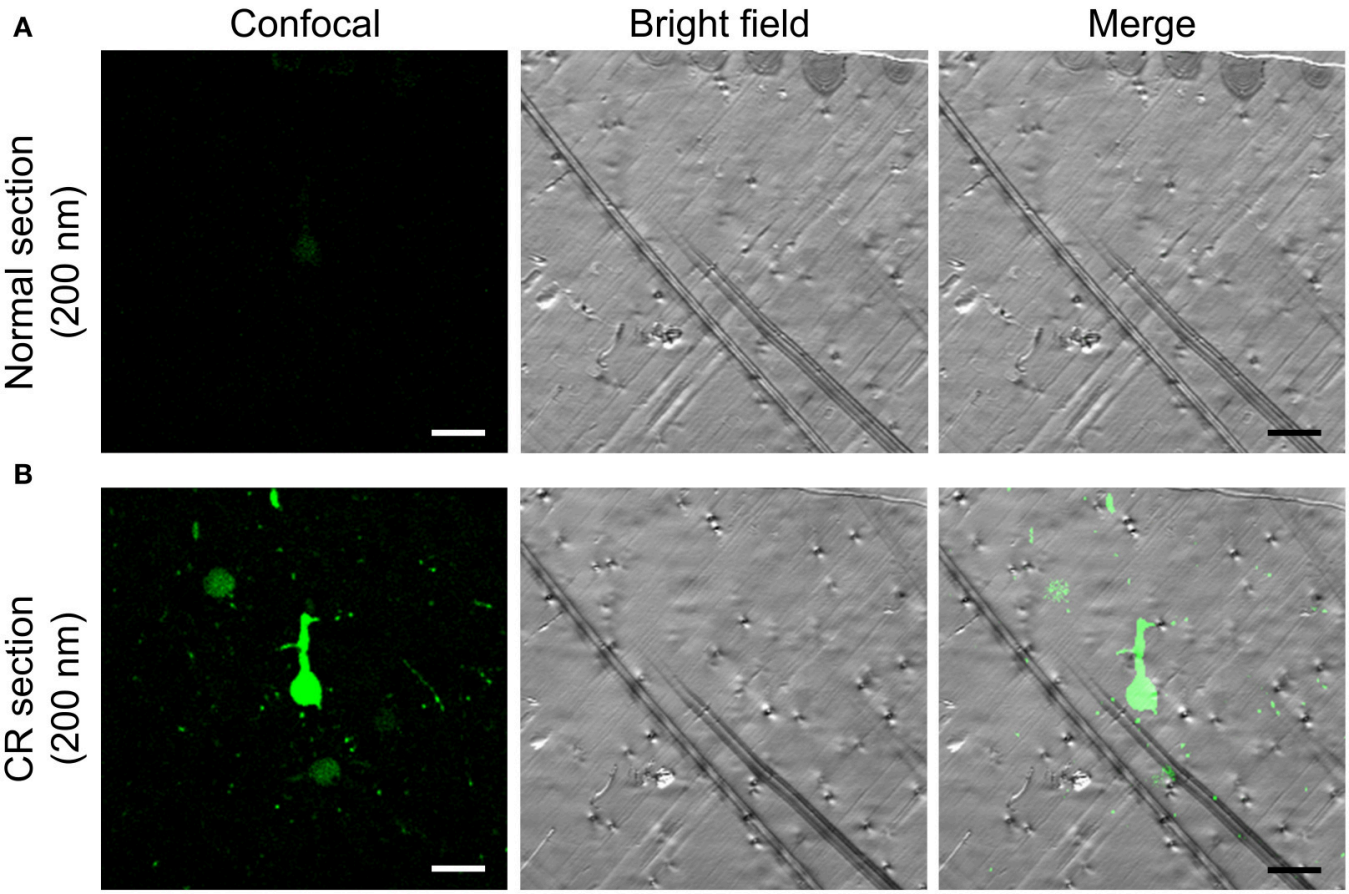
**CR in a 200 nm section from an HM20-embedded thy1-YFPH mouse brain. (A)** The normal fluorescent state of YFP brain in a 200 nm section (left), the matching bright field image (center) and the overlay (right). **(B)** The YFP fluorescence reactivated by alkaline buffer solutions in a same section (left), the matching bright field image (center) and the overlay (right) are shown. Scale bars: **(A,B)** 20 μm.

An alkali solution can rapidly penetrate resin-embedded tissue and rapidly enhance GFP/YFP fluorescence (Supplementary Video 1). For the hydrophobic HM20 resin-embedded brains, 2 min of penetration can enhance ~4 μm of the fluorescence layer. This approach can fully activate 1 μm of the fluorescence layer below the surface in 2 min (Figures 1E–G). For ultrathin (70–200 nm) section from an HM20-embedded brain, this approach can still greatly enhance fluorescence (Figure 5).

**Figure 6:**
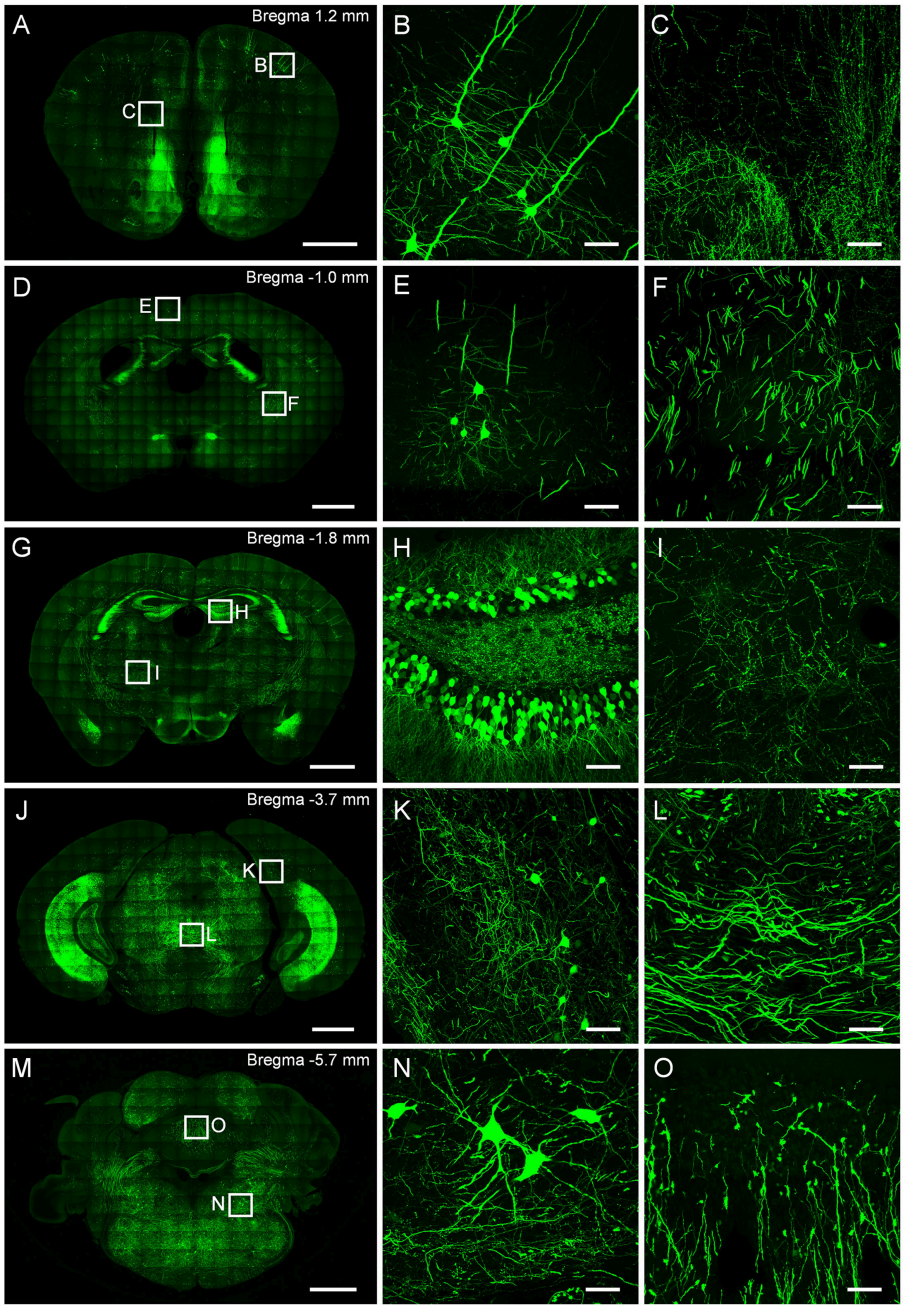
**Typical results obtained from CR of neurons and neurites in a resin-embedded thy1-GFPM mouse brain**. **(A,D,G,J)** and (m) are maximum intensity projections of different coronal plane. **(B,C)**, **(E,F)**, **(H,I)**, **(K,L)**, **(N,O)** are corresponding magnification of regions indicated in **(A,D,G,J,M)**, respectively. The projection thicknesses of **(A,D,G,J,M)** are 50 μm; **(B,C,E,F,H,I,K,L,N,O)** are 20 μm. Scale bars: **(A,D,G,J,M)** are 1 mm. **(B,C,E,F,H,I,K,L,N,O)** are 50 μm. All images were recorded on a commercial confocal microscope (Zeiss, LSM780).

**Figure 7:**
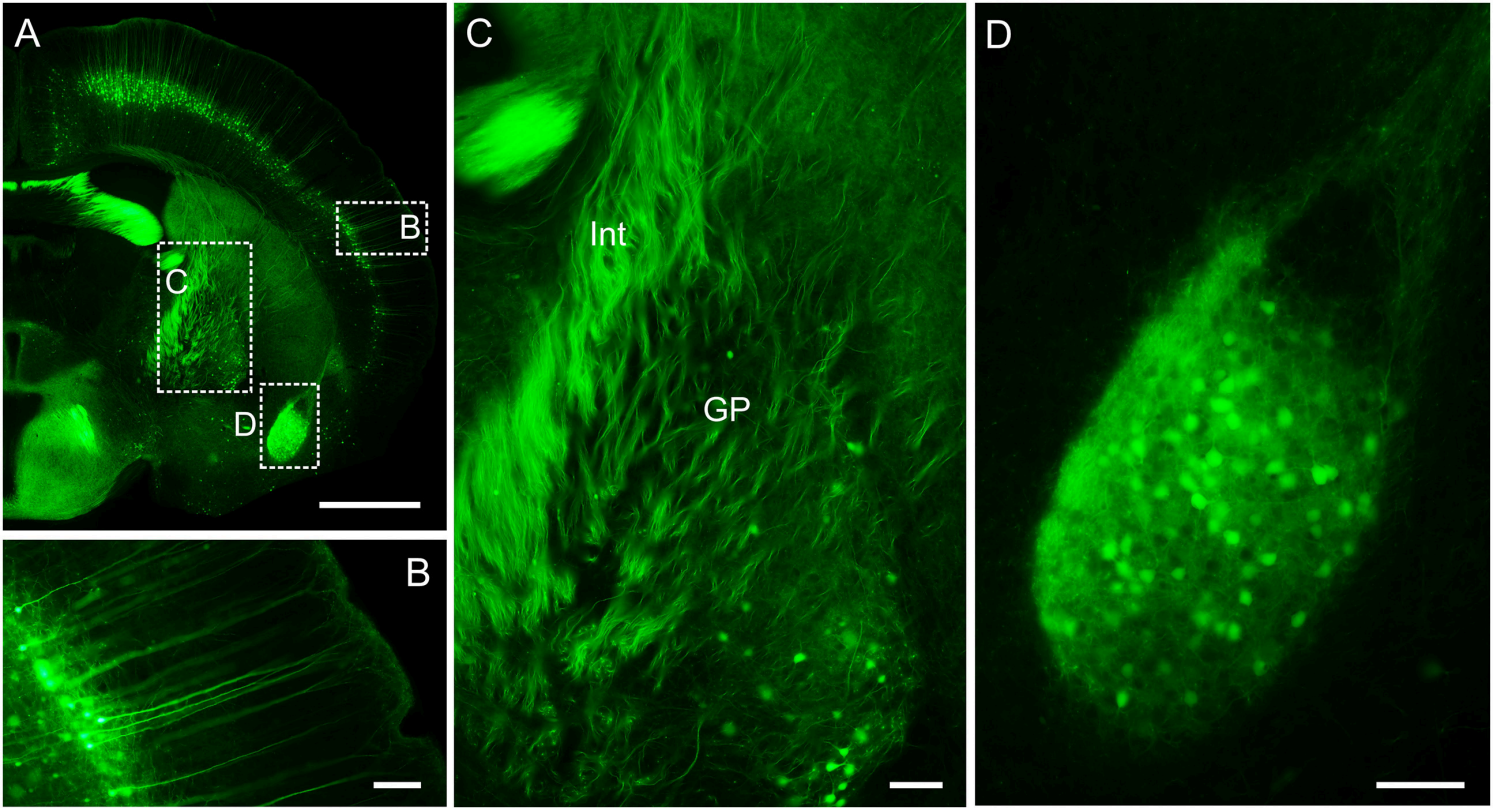
**Wide-field imaging of CR-enhanced resin-embedded thy1-GFPM mouse brain tissue. (A)** Coronal plane of a mouse brain. **(B)** Layer V pyramidal neurons in the somatosensory cortex region. **(C)** Neural fiber tracts in the internal capsule and globus pallidus regions. Int, internal capsule; GP, globus pallidus. **(D)** Neurons and axonal collaterals in the amygdaloid nucleus. Scale bars: **(A)**, 0.5 mm; **(B–D)**, 100 μm.

**Figure 8:**
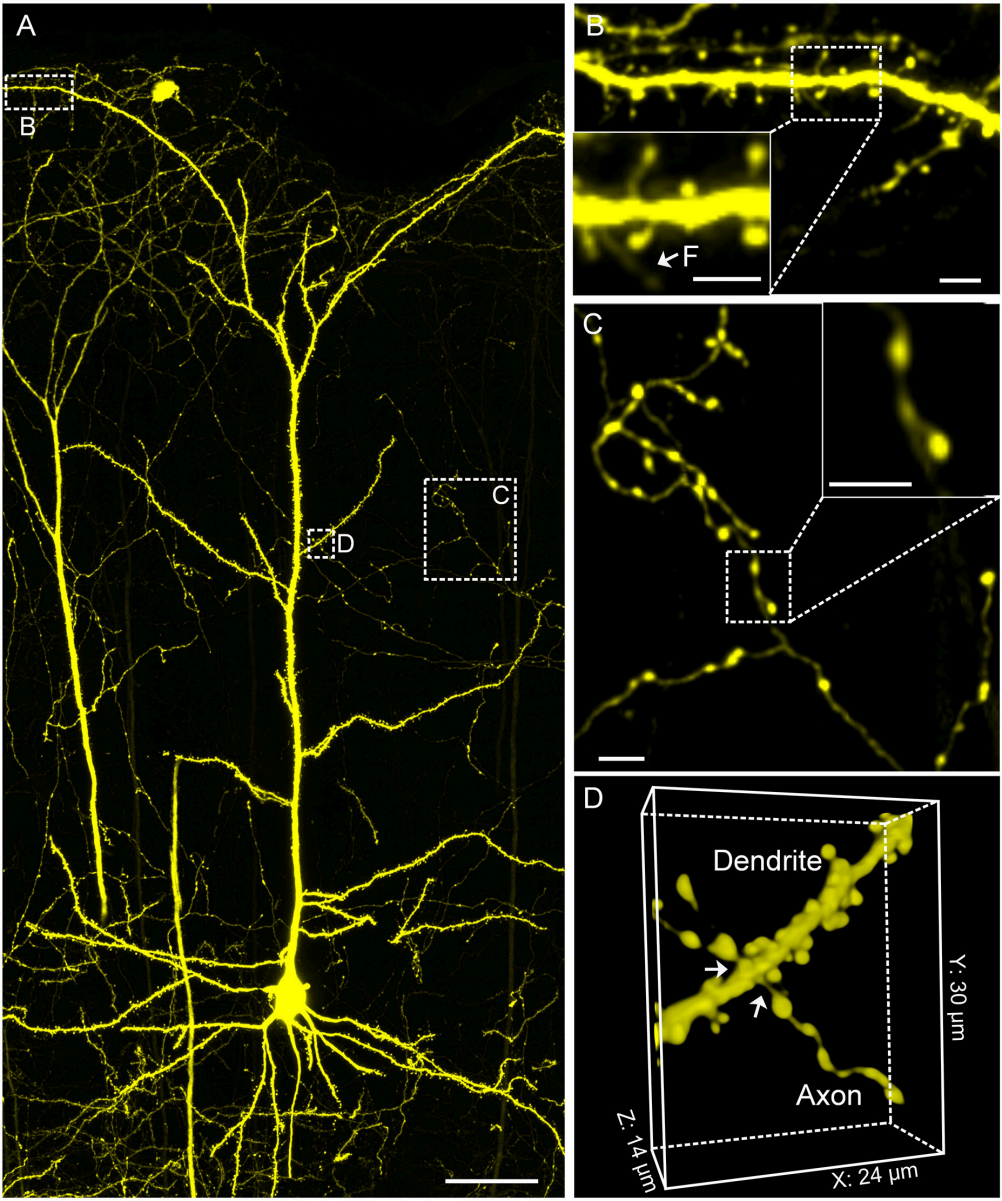
**Confocal imaging of neuronal morphology in CR-enhanced resin-embedded thy1-YFPH mouse brain. (A)** A layer V pyramidal cell. **(B)** Spine on the apical dendrite of a pyramidal cell in the cortex region. **(B)** The white arrows and F indicate the filopodium. **(C)** Structure of axon branches and axon terminal boutons on layer 3/4. **(D)** The white arrows denote junction point structures. **(D)** Junction point structure of a dendrite and axon observed under light microscopy. All images were acquired at a 0.2 × 0.2 × 1 μm^3^ voxel size on a commercial confocal microscope (Zeiss, LSM710). Scale bars: **(A)**, 50 μm; **(B), (B)** green box, **(C), (C)** green box, 5 μm; **(D)**, 24 × 30 × 14 μm^3^.

**Figure 9:**
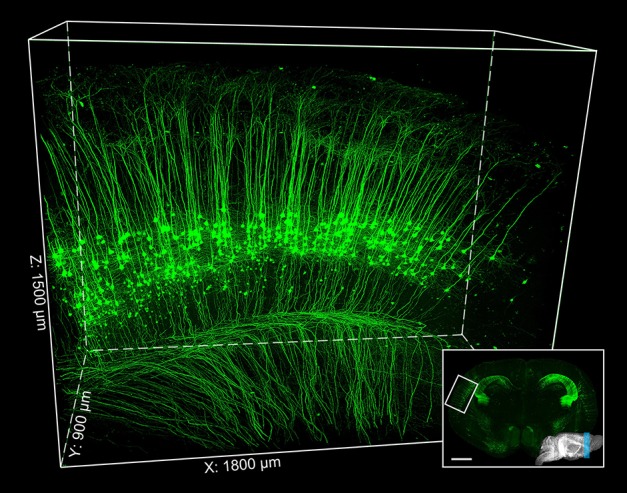
**Two-photon fMOST imaging of thy1-YFPH mouse brain**. The inset image indicates the contours of the mouse brain labeled by blue region. Parts of the cerebral cortex are 3D reconstructed (the white box region inset) and enlarged. Scale bar: inset, 1 mm; the white box region, 1800 × 900 × 1500 μm^3^.

This protocol well preserves the fluorescence signal and neural details of the whole mouse brain (Figure 6), it can be used for various optical micro-imaging technologies. Using wide-field microscopy, we can observe neurons and axonal collaterals in resin-embedded brains (Figure 7). The fine structures of neurons, such as spines, boutons, and the junction points of dendrites and axons, can be observed using confocal microscopy (Figure 8). Using two-photon fluorescence micro-optical sectioning tomography (2 P-fMOST) system, we can acquire high-resolution imaging data sets of the whole brain (Figure 9). By combining transgenic and virus labeling, we have acquired intact three-dimensional structures of specific types of neurons (Gong et al., 2016).

## Author contributions

SZ and HG initiated the current project and wrote the manuscript. YG designed and performed all of the experiments, analyzed the data, and created all of the figures and videos in addition to writing the manuscript. HZ, YJ, and LL performed the experiments and image processing. XL, QL, and XW analyzed the data. GR, XhL, LhL, HX, and ZY participated in the experiments.

### Conflict of interest statement

The authors declare that the research was conducted in the absence of any commercial or financial relationships that could be construed as a potential conflict of interest.
