# Supplementary material for: Embedding and Chemical Reactivation of Green Fluorescent Protein in the Whole Mouse Brain for Optical Micro-Imaging
Source: Front Neurosci. 2017 Mar 14;11:121. doi: 10.3389/fnins.2017.00121 (PMC5349086; doi:10.3389/fnins.2017.00121)
Supplement: Supplementary file 2 [file Presentation1.PDF]

Supplementary Material

Embedding and chemical reactivation of green fluorescent protein in the whole mouse brain for optical micro-imaging

Yadong Gang[1,2#], Hongfu Zhou[1,2#], Yao Jia[1,2], Ling Liu[1,2], Xiuli Liu[1,2], Gong Rao[1,2], Longhui Li[1,2], Xiaojun Wang[1,2], Xiaohua Lv[1,2], Hanqing Xiong[1,2], Zhongqin Yang[1,2], Qingming Luo[1,2], Hui Gong[1,2]*, Shaoqun Zeng[1,2]*

[1]Britton Chance Center for Biomedical Photonics, Wuhan National Laboratory for Optoelectronics-Huazhong University of Science and Technology, Wuhan 430074, China.

[2]Department of Biomedical Engineering, Key Laboratory of Biomedical Photonics of Ministry of Education, Huazhong University of Science and Technology, Wuhan 430074, China. Correspondence and requests for materials should be addressed to H.G. (email: huigong@mail.hust.edu.cn) or S.Z. (email: sqzeng@mail.hust.edu.cn).

[#] These authors contributed equally.

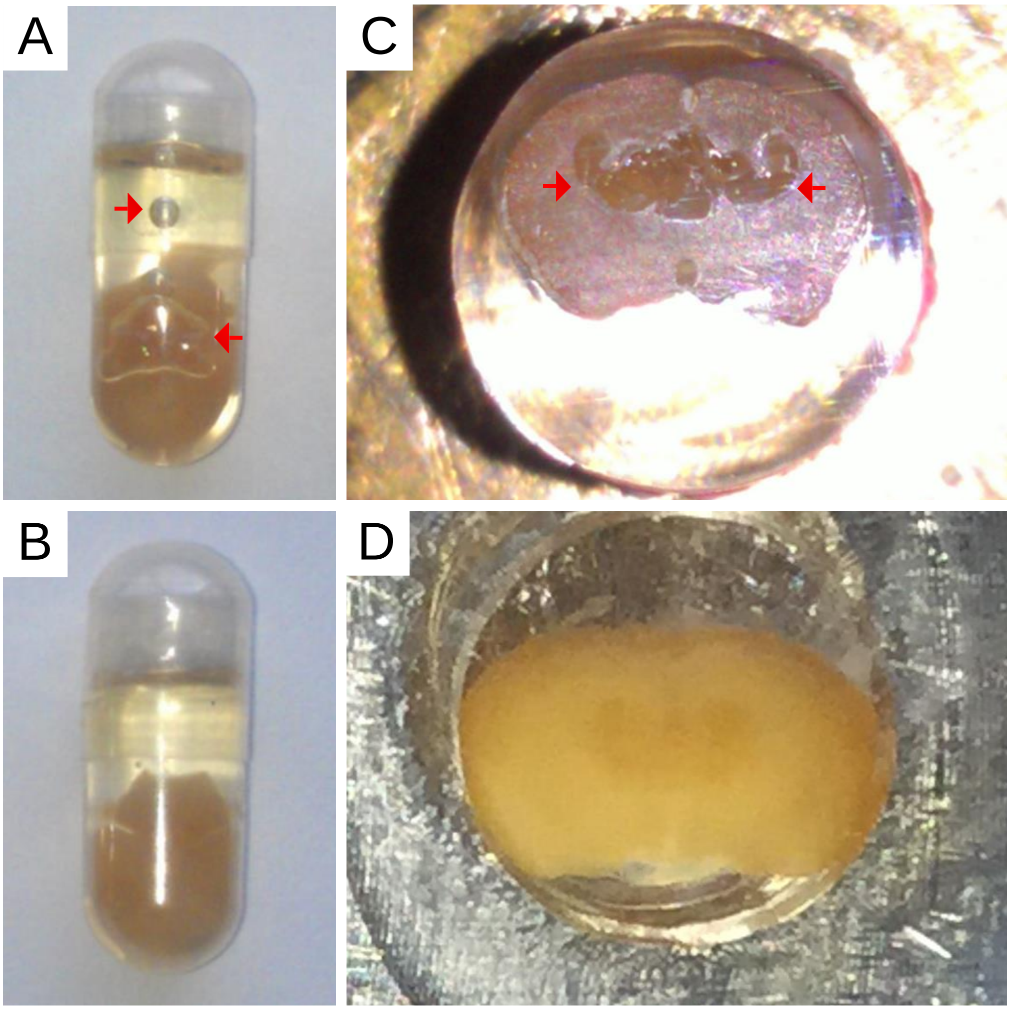

Supplementary Figure 1. Some problems in the process of dehydration and embedding. (A) Rapid polymerization generates bubbles in the sample. (B) Normal embedded sample. (C) and (D) Coronal images of anomalous and normally embedded mouse brains. In (C), a cavity in the center of sample was caused by incomplete dehydration or infiltration.

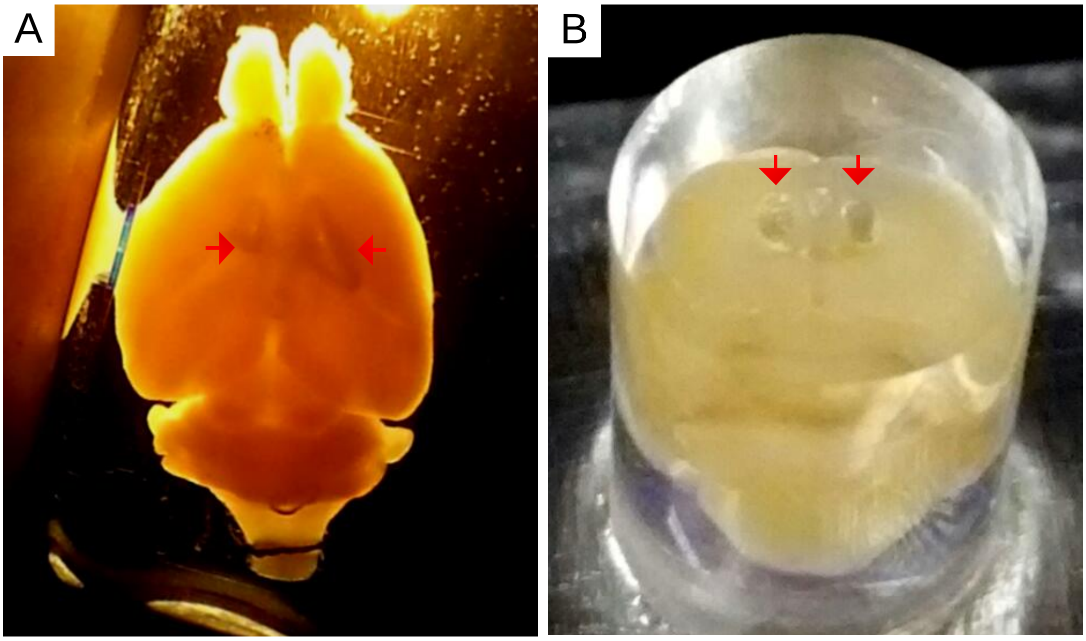

Supplementary Figure 2. Problems in the process of perfusion.
(A) Bubbles are perfused into the ventricle. (B) A cavity forms in the ventricle of a resin-embedded brain.

Supplementary Video 1. CR in a resin-embedded YFP-labeled mouse brain (Video).
